# Effect of reading with a mobile phone and text on accommodation in young adults

**DOI:** 10.1007/s00417-020-05054-3

**Published:** 2021-01-19

**Authors:** Xintong Liang, Shifei Wei, Shi-Ming Li, Wenzai An, Jialing Du, Ningli Wang

**Affiliations:** 1 Beijing Institute of Ophthalmology, Beijing Tongren Eye Center, Beijing Tongren Hospital, Capital Medical University, Beijing, China; 2 Beijing Ophthalmology & Visual Sciences Key Laboratory, NO. 1 Dongjiaominxiang Street, Dongcheng District, Beijing, 100730 China

**Keywords:** Accommodation, Mobile phone, Text, Myopic young adults

## Abstract

**Purpose:**

To investigate the effects of reading with mobile phone versus text on accommodation accuracy and near work-induced transient myopia (NITM) and its subsequent decay during near reading in young adults with mild to moderate myopia.

**Methods:**

The refractions of 31 young adults were measured with an open-field autorefractor (WAM-5500, Grand Seiko) for two reading tasks with a mobile phone and text at 33 cm. The mean age of the young adults was 24.35 ± 1.80 years. The baseline refractive aspects were determined clinically with full distance refractive correction in place. The initial NITM and its decay time and accommodative lag were assessed objectively immediately after binocularly viewing a mobile phone or text for 40 min.

**Results:**

The mean ± standard deviation (SD) initial NITM magnitude was greater for reading with text (0.23 ± 0.26 D) than for reading with mobile phone (0.12 ± 0.17 D), but there was no significant difference between the two reading tasks (*p* = 0.082). The decay time (median, first quartile, and third quartile) was 60 s (16, 154) and 70 s (32, 180) in the phone task and text task groups, respectively. There was also no significant difference in the decay time between the two reading types in general (*p* = 0.294). The accommodative lags of text tasks and mobile phones tasks were equivalent (1.27 ± 0.52 D vs 1.31 ± 0.64 D, *p* = 0.792).

**Conclusion:**

There were no significant differences in accommodative lags and the initial NITM and its decay time between reading with a mobile phone and text in young adults.

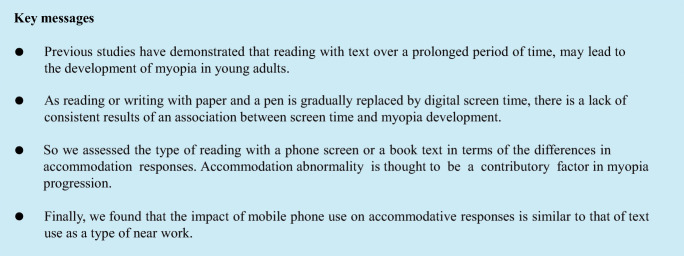

## Introduction

Myopia has become an important public health issue worldwide, especially in Asian countries, and is a major cause of correctable visual impairment [1, 2]. The increasing prevalence of myopia is thought to be linked to the environment, such as intensive education, more near work, and less time spent outdoors [3–8]. Several studies have reported that near work is a vital contributor to myopia [9, 10], but the exact reasons why near work exacerbates myopia are not well understood. This has attracted the interest of many researchers, and they have tried to determine the possible mechanisms by which periods of prolonged near work could result in myopia. Different mechanisms have been found to be proposed, including accommodation error during near work (which means a lag in accommodation when the accommodation response is unable to meet the dioptric demand) and the small transient myopic far point shift immediately after long-term near work [3, 11]. These two mechanisms are thought to be key factors in myopia progression.

Near work-induced transient myopia (NITM) is related to a myopic accommodative aftereffect that is caused by an inability of the crystalline lens to recover its power to distance refractive status appropriately and rapidly after continuous near work [12]. NITM can be influenced by near task duration and the refractive group [13]. Several studies have shown that adult myopes exhibited increased initial magnitude and decay duration of NITM compared with emmetropia and hyperopia groups [14–16]. It has been thought that NITM may be related to permanent myopia, but whether differences in NITM are a cause or consequence of myopia cannot be answered [17]. Currently, many studies have paid attention to lags in accommodation during and after prolonged near work in different refractive groups [14, 18]. Both myopic adults and children exhibit lags in accommodation during near work. One important finding is that there is an obviously increased lag in accommodation in progressing myopes when compared with emmetropes and stable myopes [16, 19]. Therefore, accommodative lag is also thought to be a contributor to myopia development.

With the development of digital devices in recent years, computers, smart phones, and tablets have been argued to play a role in myopia progression. Thus, children are now exposed to another potential environmental risk factor for myopia—digital screen time [20]. Some previous studies showed that the use of computers was significantly associated with the prevalence of myopia and that there was more myopic refractive error in children 5–16 years old [21–25]. However, there is a lack of consistent results of an association between screen time and myopia development. Several studies reported that the number of hours per day playing with electronic devices was not associated with myopia [26–28]. Therefore, this is a current issue. With the increasing shift from printed to electronically produced text over the past three decades, many previous studies of NITM were carried out in the late 1990s or early 2000s using printed text accommodative stimuli, so it is important to see if the shift to electronic displays has altered the accommodative behavior. Our study wanted to examine whether there is a difference between reading with a digital screen and a traditional book text on accommodation responses and myopia. The type of reading with a phone screen or a book text has not been assessed in terms of the differences in accommodation responses.

Therefore, the aim of this study was to investigate the effect of reading with a mobile phone and book text on the magnitude of NITM and its decay time and accommodative lags. We also wanted to determine whether reading with a phone or text had different effects on NITM and accommodative lags. We hypothesized that there was no significant difference in NITM and its decay time and accommodative lags for reading with text and a mobile phone.

## Methods

### Subjects

Thirty-one subjects (13 males and 18 females) between the ages of 20 and 27 years were recruited from the Capital Medical University in China. This research followed the tenets of the Declaration of Helsinki and was approved by Beijing Tongren Hospital Ethics Committee. Informed consent was obtained from each subject. All the subjects met the inclusion criteria of full-time spectacle wearers to avoid the influence of changes in refractive correction methods on normal accommodation behavior, free of significant ocular history or pathology, no accommodative anomalies, best-corrected visual acuity of at least 6/6, ≥− 6.00 D of myopia, ≤ 0.75 D of astigmatism, and ≤ 1.00 D of anisometropia, no history of ocular surgery or ocular trauma, and no severe medical or ocular health problems or mental disease, no medications use that are known to impact accommodation. All included subjects had a complete eye examination including refraction, binocular, and ocular health status. The right eye was the tested eye for all measurements.

Subjective refraction was performed using the maximum plus for best visual acuity principle [29]. Myopic refractive error was corrected for distance viewing with spectacles based on prescriptions of optometry to within ± 0.25 D (best sphere) and small astigmatic errors were corrected too. Subjects were asked to wear corrected spectacles for the reading tasks and accommodation measures. Each subject completed two reading tasks involving text and a mobile phone.

### Procedures

Two reading tasks were provided in random order to each subject: reading with a mobile phone and text. We adopted 33 cm as the reading distances for both tasks. All the subjects were provided with the same mobile phone and book by our research group, and the reading content consisted of paragraphs of text with a difficulty level appropriate to the education level of the adults. Both types of texts were darker letters on a lighter background and composed of paragraphs of words in 10.5-point Sim Sun Chinese font with an environmental luminance of 100 cd/m^2^. The print size of the book was the same as A4 paper and the contrast of the text was 90%. The phone model we used was HUAWEI and the size of the phone screen was approximately 15.0 × 7.3 cm with a luminance of 80 cd/m^2^. The resolution of this screen was 2244 × 1080 dpi. Luminance was measured using the Topcon luminance colorimeter BM-7. The Michaelson contrast of the text was calculated using the formula (L max − L min) / (L max + L min), in which L max is the maximum luminance of the lighter background and L min is the minimum luminance of the letters [11].

An open-field autorefractor (WAM-5500; Grand Seiko Co., Ltd., Hiroshima, Japan) was used to measure the refractive state before and after the 40-min reading period. This device has been demonstrated to be reliable for NITM and its decay and accommodative stimulus-response testing in its dynamic mode [30]. The precision of this dynamic mode was 0.01 D per the manufacturer’s manual. The dynamic measurements were taken at approximately 5 Hz (five samples per second). A computer was connected to the Grand Seiko WAM-5500 autorefractor using an RS-232C cable to collect the high-speed measurements and the data output can be automatically converted to an Excel spreadsheet.

All the subjects were asked to stay in a completely dark room for 5 min to relax the accommodative spasms. Then, the lights were switched on, and the participants were seated in front of the autorefractor under fluorescent illumination. Monocular refractive measurements were then performed on right eyes using the autorefractor while subjects were asked to view the distance target of 20/30 Snellen letters at 4 m with spectacle lens refractive correction in place. Fifty measurements at 0.2-s time intervals were made. The mean spherical equivalent (SE) was calculated from these data, representing the mean baseline distance refractive state. Next, the subjects performed one of the reading tasks for 40 min at a 33-cm distance. The reading distance was measured every 5 min by the examiner to ensure its consistency during the task [31]. Moreover, the subjects were asked to view the words as clearly as possible at all times. Posttask accommodation response measurements were immediately made after completion of the 40-min reading task. The subjects were instructed to view the upper line of best corrected visual acuity of a test near vision chart at the fixed 33-cm distance over a period of 10 s with their chin on the chinrest of the autorefractor, and accommodation responses were assessed monocular using the Grand Seiko WAM-5500 autorefractor. Then, the subjects were asked to focus immediately on the distant Snellen target at 4 m again, and the distance refraction measurements were obtained continuously for a period of 180 s (900 measurements). The average of the first 10-s measurement magnitudes represented the posttask refraction. The time taken for the posttask refraction to dissipate to the average pretask refraction and be maintained for at least 10 s was thought to be the decay time.

In between the two reading tasks, the subjects took at least 10 min of a break in a completely dark room.

### Data analysis

After data collection, the mean SE was averaged of all the measurements for 10-s bins in the right eye. The magnitude of NITM was calculated by subtracting the mean SE of baseline distance refraction measurements from posttask distance refraction measurements. Initial NITM was calculated during the first 10-s interval. The time taken for the posttask refraction to reach to the average pretask refraction and be maintained for at least 10 s was thought to be the decay time. If the accommodation had not dissipated to pretask levels by 3 min, it was classified as incomplete decay NITM, and a value of 180 s was recorded. The accommodative lags were determined by subtracting the accommodative response measurements from the accommodation demand. Spectacle lens correction will lead to variation in the accommodation demand and magnification with the magnitude of refractive correction change. Therefore, the calculation of the accommodative demand and accommodative response was adjusted as they were affected by the presence of the lens in front of the eyes [32].

Statistical analysis was performed with the Statistical Program for the Social Sciences (IBM SPSS Statistics 25). The independent-sample *t* test was carried out to determine the significance of the within-subject task variable of reading type (mobile phone and text) and between-group factor of refraction error (mild myopes, moderate myopes) on initial NITM and the accommodative lags. The decay time of NITM was analyzed using the nonparametric Kruskal-Wallis test. A value of *p* < 0.05 was considered to be statistically significant.

## Results

The mean age of all subjects was 24.35 ± 1.80 years with a mean spherical equivalent of − 3.18 ± 1.54 D (range, − 5.75 D to − 0.88 D). The results of initial NITM and its decay time and the accommodative lags with two different reading types are summarized in Table 1. The effect of reading types and refractive error group is described in detail subsequently.

**Table 1:** Mobile phone versus book text: accommodative lags, initial NITM, and decay time

Measures	Period	Book text	Mobile phone	*P*
Accommodative lags (D)	Posttask	+ 1.27 ± 0.52	+ 1.31 ± 0.64	0.792
NITM (D)	First 10 s	+ 0.23 ± 0.26	+ 0.12 ± 0.17	0.082
Decay time, s, (median, first quartile, and third quartile)	To pretask level	70 (32, 180)	60 (16, 154)	0.294

Spherical equivalents are presented as the mean ± SD in diopters

Number of data excludes abnormal magnitudes due to measurement errors

### Effect of reading type and refractive error on initial NITM and its decay time

**Fig. 1:**
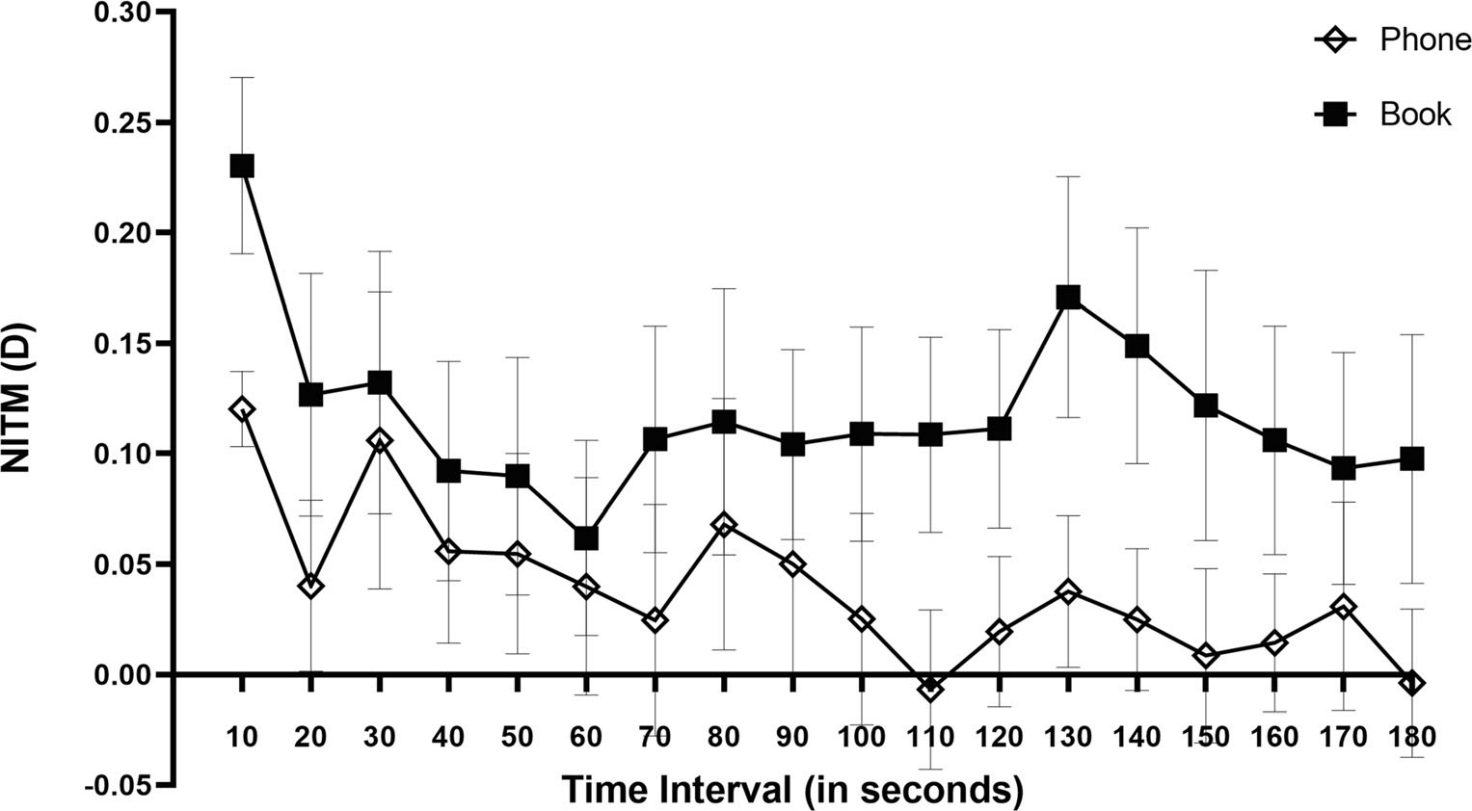
Decay of nearwork-induced transient myopia (NITM) as a function of refractive state for the two reading types. Plotted is the mean ± SEM

**Fig. 2:**
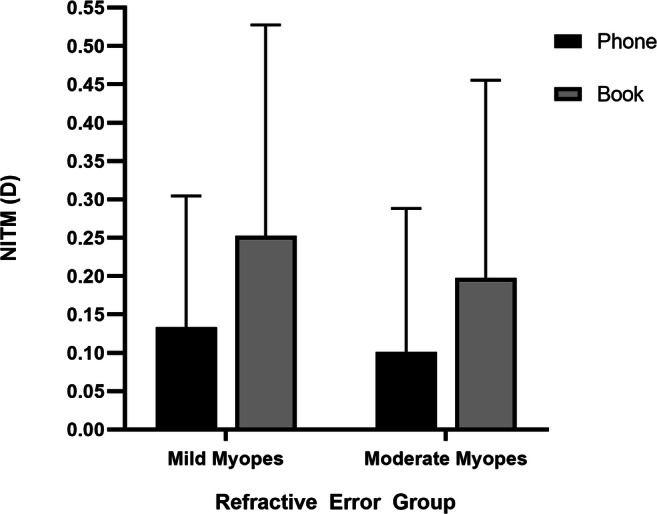
Effect of two reading types on the initial NITM in the mild and moderate myope groups. Data are reported as the mean ± SD

There were 31 subjects in total, 27 datasets for NITM were effective (2 subjects were missing the data for posttask distance refraction measurements and 2 subjects had abnormal magnitudes due to measurement errors) in the text task. In the phone task, 25 of 31 datasets were effective (3 subjects were missing data for posttask distance refraction measurements and 3 subjects had abnormal magnitudes due to measurement errors). Among these data, 25/27 and 22/25 NITM magnitudes had an effective decay time in the text task and phone task, respectively (5 datasets were excluded due to eye movement during measurement). The initial NITM (mean ± SD) was greater for the reading with text tasks (+ 0.23 ± 0.26 D) than for the reading with mobile phone tasks (+ 0.12 ± 0.17 D) and took 10 s longer to dissipate in the text task, but there was no significant difference between the mobile phone and text groups in the initial NITM (*P* = 0.082) and decay time (*P* = 0.294). Figure 1 presents the average NITM decay for subjects with complete and incomplete decay during the 3-min posttask interval as a function of refractive state. The decay profile was reading type dependent. The text group exhibited relatively higher NITM, with incomplete decay during the entire 3-min posttask period. Thus, the data were analyzed with different refraction groups included as the between-group factor including mild myopes (SE between − 0.50 D and − 3.00 D) and moderate myopes (SE between − 3.00 D and − 6.00 D). They were further classified as 16 mild myopes and 11 moderate myopes in text group. In the phone group, there were 15 mild myopes and 10 moderate myopes. Figure 2 shows the mean initial NITM and their standard deviations for each reading type and the two refractive groups. The mean initial NITM and standard deviations for the mobile phone task were + 0.13 ± 0.17 D for the mild myope group and + 0.10 ± 0.19 D for the moderate myope group, and the mean for the text task was + 0.25 ± 0.27 D for the mild myope group and + 0.20 ± 0.26 D for the moderate myope group. The initial NITM of the text task was greater than that of the phone task both in the mild myope and moderate myope groups, but no significant difference was found between the phone task and text task either in the mild myope group (*p* = 0.158) or in the moderate myope group (*p* = 0.340).

### Effect of reading type and refractive error on accommodative lags

**Fig. 3:**
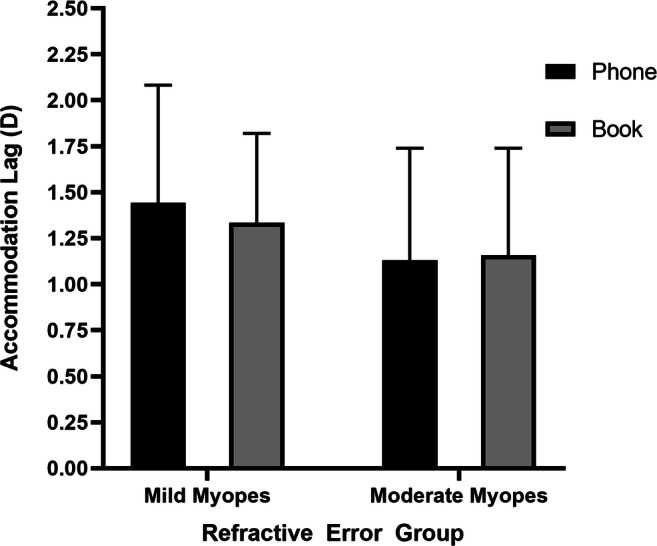
Effect of two reading types on accommodation lags in the mild myope group and moderate myope group. Data are reported as the mean ± SD

There were 30/31 subjects from the phone task group and 29/31 from the text task group with complete data of accommodation responses, including both pretask and posttask data. As Table 1 shows, in general, the phone task (+ 1.31 ± 0.64) showed slightly larger accommodation lags than the text task (+ 1.27 ± 0.52), but this difference was not significant (*P* = 0.792). The subjects were further classified as 18 mild myopes and 11 moderate myopes in text group. In the phone group, there were 17 mild myopes and 13 moderate myopes. Figure 3 shows the mean accommodative lags of the two refractive groups for each reading type. The mean accommodative lags for the phone task were + 1.44 ± 0.64 D for the mild myope group and + 1.13 ± 0.61 D for the moderate myope group, and the mean for the text task was + 1.34 ± 0.48 D for the mild myope group and + 1.16 ± 0.58 D for the moderate myope group. The accommodative lags of the phone task were greater than those of the text task in mild myopes; in contrast, the accommodative lags of the text task were greater than those of the phone task in the moderate myope group, but no significant difference was observed between the phone task and text task either in the mild myope group (*p* = 0.576) or in the moderate myope group (*p* = 0.915).

## Discussion

In our study, the findings support our hypothesis that reading with text and a mobile phone had no significant difference on NITM and its decay and accommodative lags (*P* > 0.05).

Many previous studies have investigated the association between near work and myopia and demonstrated that near work is related to myopia [33–35]. Konstantopoulos et al. [36] found that near work, such as reading over a prolonged period of time, may lead to the development of myopia in young adults. To better understand the mechanism of near work and its possible relation to myopia, many clinical studies have been conducted. For example, Vera-Diaz et al. [16] reported that the increase in NITM and a slow NITM decay time were involved in the progressive phase of myopia. Abbott et al. [19] measured the accommodation stimulus response curves of adult subjects during near work and provided further evidence that the lags in accommodation responses occur mainly during the progression of myopia. This has resulted in support for the initial NITM magnitude and its decay and accommodative lags as key factors for myopia development after a sustained near work task.

Epidemiological studies, such as the Sydney Myopia Study (SMS) [27], the Singapore Cohort of Risk factors for Myopia (SCORM) [37], and the Anyang Childhood Eye Study (ACES) [38], have shown the relationship between reading and myopia progression. The SMS found that close reading distance (< 30 cm) and continuous reading (> 30 min) independently increased the odds of having myopia. The SCORM study reported that reading more than two books each week was associated with more negative refractive errors. The ACES showed that a close reading distance (< 20 cm) and reading lasting for more than 45 min without a break were significantly associated with myopia. However, with the development of modern technology, electronic devices are more and more popular among young adults, and this equipment is an important part of their everyday lives. Thus, students are used to acquiring knowledge and other information by using smart phones. Hence, it is important to investigate the relationship between smart phone and myopia. Smart phones and traditional books are both a constitutive part of near work, but the phone screens differ from traditional books in aspects such as their color, brightness, contrast, and resolution. Therefore, this study compared the effects of traditional books and phones on NITM and its decay and accommodative lags to show if smart phones are associated with myopia progression.

Several studies have confirmed that using electronic devices can increase the risk of myopia progression. For example, a previous study found that using a computer more than 3 h per day was a high-risk factor for myopia in university students [39]. Liu et al. [40] supported that the more time individuals spent using smart phones and computers, the more myopic equivalent spherical refraction and longer axial length they had. A large longitudinal assessment in young adults was conducted by Fernández-Montero et al. [41] and showed that exposure to computer use is associated with myopia development in a cohort of Spanish university graduates. However, the relationship between electronic devices and myopia has had mixed findings. A review written by Carla et al. [20] showed that the prevalence, incidence, or progression of myopia was not clearly associated with screen time. In our study, we observed the magnitudes of NITM and its decay and accommodative lags in reading with a mobile phone and text, and there were no significant differences between book and phone use. The results provide evidence that reading with a mobile phone and text has similar effects on the accommodation response, and both can increase the risk of myopia progression. Our research suggested that mobile phone use may not be a causal factor but may be a replacement for a different type of near work. We provided data to tease out mobile phone use as a proxy of near work time in young adults because it has a similar effect on accommodation responses as reading with text. In recent education, digital screen time is likely to be substituted for traditional reading or writing with paper and a pen. In recreation, mobile phones are mainly used to play electronic games and watch videos. Dirani et al. [42] believe that the increased time spent on digital devices revealed more near work and less outdoor activity, resulting in myopia progression. Therefore, it is important to pay more attention to restrict the use time of digital devices.

One of the limitations of our research was the setting of the reading and testing tasks. When the subject switched to the measurement task for accommodative lags from the reading task, it would take 1 to 2 s to place their chin onto the chinrest of the autorefractor and thus resulted in an inevitable loss of fixation on the 33-cm target. Another limitation was that the reading time was only 40 min, and further investigations are required to know if the differences in accommodation will occur between the reading with text and phone groups following longer or shorter reading periods. Therefore, the differences in reading time may then become important. The other limitation was that all the subjects were young adults, and the accommodative fluctuations of young adults are smaller than those of children [43], resulting in differences that were not significant between the phone and text tasks. Last but not least, all the subjects had mild to moderate myopia, and those with emmetropia may have different results, but our study did not include those with emmetropia.

## Conclusion

In summary, reading with text tasks caused a larger NITM, longer decay time, and greater accommodative lags than reading with a mobile phone, but the small differences were not clinically significant. Current evidence suggests that the impact of mobile phone use on accommodative responses is similar to that of text use as a type of near work. As conventional educational reading or writing with paper and a pen is gradually replaced by digital screen time and with more recreational screen time in adolescents, effective measures should be taken to limit the use time of digital devices.

## Data Availability

All data generated or analyzed during this study are included in this published article.

## Abbreviations

NITM: Near work-induced transient myopia
SMS: The Sydney Myopia Study
SCORM: The Singapore Cohort of Risk factors for Myopia
ACES: The Anyang Childhood Eye Study
SD: Standard deviation
